# “Expert persuasion” can decrease willingness to pay for sugar-containing food

**DOI:** 10.3389/fnut.2022.926875

**Published:** 2022-07-28

**Authors:** Ioannis Ntoumanis, Ksenia Panidi, Yaroslava Grebenschikova, Anna N. Shestakova, Vladimir Kosonogov, Iiro P. Jääskeläinen, Dzerassa Kadieva, Sofia Baran, Vasily Klucharev

**Affiliations:** ^1^International Laboratory of Social Neurobiology, Institute of Cognitive Neuroscience, HSE University, Moscow, Russia; ^2^Department of Psychology, Institute of Cognitive Neuroscience, HSE University, Moscow, Russia; ^3^Brain and Mind Laboratory, Department of Neuroscience and Biomedical Engineering, Aalto University School of Science, Espoo, Finland

**Keywords:** food choices, healthy eating, willingness to pay, narratives, sugar, need for cognition, diet and health knowledge, expert persuasion

## Abstract

Recent studies have revealed types of eating nudges that can steer consumers toward choosing healthier options. However, most of the previously studied interventions target individual decisions and are not directed to changing consumers’ underlying perception of unhealthy food. Here, we investigate how a healthy eating call—first-person narrative by a health expert—affects individuals’ willingness to pay (WTP) for sugar-free and sugar-containing food products. Participants performed two blocks of a bidding task, in which they had to bid on sweets labeled either as “sugar- free” or as “sugar-containing.” In-between the two blocks, half of the participants listened to a narrative by a dietary specialist emphasizing the health risks of sugar consumption, whereas the remaining participants listened to a control narrative irrelevant to food choices. We demonstrate that the health expert’s narrative decreased individuals’ WTP for sugar-containing food, but did not modulate their WTP for sugar- free food. Overall, our findings confirm that consumers may conform to healthy eating calls by rather devaluating unhealthy food products than by increasing the value of healthy ones. This paves the way for an avenue of innovative marketing strategies to support individuals in their food choices.

## Introduction

Inside a grocery store, we are often surrounded by high-calorie, sugary food which is just irresistible. In such obesogenic environments we live in, individuals need support to maintain their healthy eating goals, especially given the increasing obesity rates. However, what can actually influence individuals toward a healthy diet? This has become a central question in consumer research (1–7). Although there is a great amount of studies investigating the food choice determinants (8–11) and the effectiveness of various health nudge interventions (12, 13), there is limited research on whether and how nudges can affect sugar consumption. A recent meta-analysis (12) showed that *healthy eating calls*, written or oral injunctions to reduce unhealthy choices, reliably reduce unhealthy eating. Here, we suggest an experimental paradigm that allows us to further investigate how a healthy eating call by a health expert affects individuals’ willingness to pay (WTP) for sugar-free and sugar-containing food products.

Earlier studies investigating sugar-related nudge interventions have applied either visibility enhancements or labels and the results are inconsistent. Specifically, Shin and Kim (14) showed that displaying less sugary beverages at eye-level shelf position increases consumers’ demand for the beverages. Moreover, reduced-sugar labels have been associated with higher WTP, but also with lower perceived healthfulness and tastefulness (15, 16). In contrast, Drugova et al. (17) showed that people are willing to pay less for products labeled as sugar-free. On the other hand, some of the reduced-sugar labels that have been tested in the past, have revealed null effects (18–20). Importantly, in most of these experiments the sugar- related labels were displayed together with additional labels or claims raising concerns about whether the observed effects are confounded by information not related to sugar. Besides, given that visibility enhancements and labels have been found to be the least effective types of nudging (12), it is important to further study more effective nudges including healthy eating calls such as first-person narratives by health experts.

The effect of narratives on decision-making is well documented both at the behavioral (21) and at the neural level [for a review see (22)]. However, there is a limited number of studies investigating the effect of narratives on food evaluation, and most of them are limited either to children populations or to subjects’ evaluation of a single product (23–27). We consider this an important gap in the field, since a detailed narrative about unhealthy eating would have a great potential to affect not only individual decisions, but also people’s underlying perception of unhealthy food. First-person narratives, in particular, can have properties of both injunctive and descriptive norms through the opinion and behavior of the narrator, and social norms influence food choices (11, 28–30). Notably, we use the term *narrative* in a general manner, referring to the entire “rhetorical tetrad” of (31), i.e., including arguments that might be personal or impersonal and general or particular.

If first-person narratives were to be used as healthy eating calls, the narrator would have to be carefully selected, since their credibility is likely to moderate the narrative’s effectiveness. In general, communicators with high expertise are particularly persuasive (32–35), and this phenomenon is also observed in food decision-making (24, 36, 37). Hence, in the current study, the narrator was introduced to the experimental participants as a dietary specialist, to make the healthy eating call maximally persuasive. Moreover, personality traits have been found to shape processing of narratives in the brain (22, 38, 39), as well as to moderate the behavioral effect of narratives on decision- making (40). Food choices are also influenced by individual differences in personality, and so does the susceptibility to nudges (41–43). Therefore, in the current study we examined the potential moderating role of participants’ need for cognition (NFC) and health knowledge in the effect of narratives on food choices. We pinpointed these particular traits, because their moderating role in the effect of narrative messages on food evaluation has been highlighted (26). NFC has also been found to moderate the persistence and resistance of attitude changes (44).

First, we hypothesized that, after listening to a first-person healthy eating call emphasizing the risks of sugar consumption, individuals would decrease their WTP for sugar-containing products, since previous studies suggest that interventions are more effective at reducing unhealthy eating than increasing healthy eating (12). Second, we anticipated that the effect of the health expert’s narrative on food valuation would be pronounced in people with high NFC and low health knowledge. Because of the putative cognitive effort needed to process the scientific content of the health expert’s healthy eating call, we expected that individuals with high NFC would be more engaged in it. Subsequently, their behavior would be modulated to a larger extent as compared to those with low NFC. Moreover, individuals with high health knowledge were likely to be aware of the sugar consumption risks even before listening to the narrative, making them less susceptible to the narrative’s message as compared to those with low health knowledge. Importantly, to our knowledge no study to date has investigated the effect of narratives by health experts on sugar consumption.

## Materials and methods

### Participants

Forty-eight participants were recruited to participate in the experiment (60% females, aged 18–44 years, mean age = 24.75). They were randomly and evenly allocated to either the experimental or the control group. The two groups did not differ in age (*t* = 0.366, *p* = 0.716) or educational level (Pearson’s chi-squared test of homogeneity, χ^2^(3, 48) = 0.277, *p* = 0.964). All of them were right-handed, healthy, had normal or corrected-to-normal vision, had no history of psychiatric diagnoses or eating disorders, no neurological or metabolic illnesses, and were not taking any prescribed medication. All participants mentioned that they eat sweets in general. Participants were told that the goal of the experiment was to study food preferences. On top of the reward based on their decisions in the bidding task, all participants received a flat fee of 150 monetary units (MU) equivalent to ∼$5.7, with the correction for purchasing power parities (45).

The food stimuli and the healthy eating call used in this study were validated by three independent cohorts. Twelve participants (7 females, ages 19–53 years, mean age = 31 years) were recruited to indicate their WTP and their perceived tastefulness, sweetness and healthfulness of the food stimuli. Twenty participants (15 females, aged 19–45, mean age = 24) were recruited to assess the emotional effect of the experimental narrative. Twenty-nine participants (24 females, aged 18–26 years, mean age = 20.65) were recruited for another experiment, where they rated their emotions after listening to the control narrative used in the present study.

### Materials

#### Food stimuli

**FIGURE 1:**
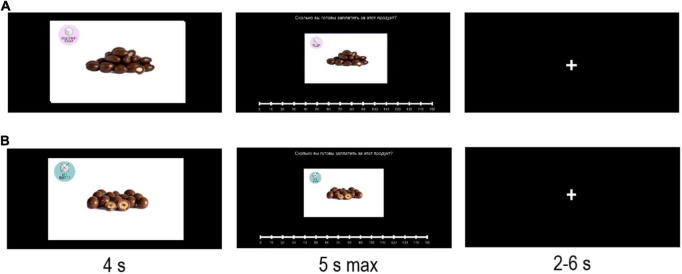
Bidding task. Sample trial with food labeled as sugar-containing **(A)** and sample trial with food labeled as sugar-free **(B)**. At the beginning of each trial, a product was displayed for 4 s. Next, a message was displayed at the top of the screen “How much does this product worth to you?” as in (66). Participants had 5 s to indicate their WTP for this product, using a discrete slider (0–150 MU, with an increment of 10 MU). Last, a fixation cross was shown (2–6 s) and the next trial began.

Sixty full-colored photographic pictures (200 dpi) of food products were used, half of which were labeled as “sugar-free” and half as “sugar-containing.” Participants were told that the “sugar-free” label indicates that the product does not contain sugar. The labels were not deceptive. All products existed in the market during the period of data collection. The pictures represented food without packaging (Figure 1). The category (e.g., cookies), the size and the actual price of the products were counterbalanced across conditions. Moreover, we pre-tested the WTP and the perceived sweetness, tastefulness and healthfulness of all products without using labels and the average ratings were not significantly different between sugar-free and sugar-containing foods (Supplementary Table 1). In addition, prior to data collection we conducted a survey to examine people’s opinion about refined sugar and products labeled as “sugar-free” (Supplementary Table 2). We also presented the labels with different colors, so that participants could better distinguish the two conditions. Specifically, half of the participants were presented with the sugar-containing label in blue and the sugar-free label in pink, and the other half of the participants vice versa.

#### Bidding task

Figure 1 illustrates the procedure in the bidding task. At the beginning of each trial, a product was displayed for 4 s. Afterward, participants had 5 s to indicate, with the mouse, their WTP in order to purchase this product at the end of the experiment, using a slider (0–150 MU, with an increment of 10 MU), following previous studies (4, 46, 47). If a participant did not respond within the time limit, the WTP was set to 0 MU (46). Each block contained the same amount of “sugar-free” and “sugar-containing” products and the order of the items was randomized across participants and blocks. Finally, a fixation cross was shown (2–6 s) and the next trial began.

The range of the prices among which participants could bid was chosen based on the range of the actual prices of the products in the market. Participants received 150 MU endowment at the beginning of the experiment, which they could use for purchasing products. The endowment was equal to the maximum WTP, so that participants did not have to worry about distributing their 150 MU over the different food products and they could treat each trial as if it were the only decision that counted (47). The Becker-DeGroot-Marschak auction was used in order to measure individual preferences and each participant’s exact WTP for every product (47, 48). Specifically, at the end of the experiment, one trial was randomly selected. Let *b* denote the bid made by the participant in that trial. A random number *n* was also drawn from a known distribution (in our case, 0, 10, …, 150 MU were chosen with equal probability). If *b* ≥ *n*, the participant received the food product corresponding to that trial and paid a price equal to *n*. Otherwise, the participant did not receive the food but also did not pay anything (47).

#### Healthy eating call and control narratives

**FIGURE 2:**
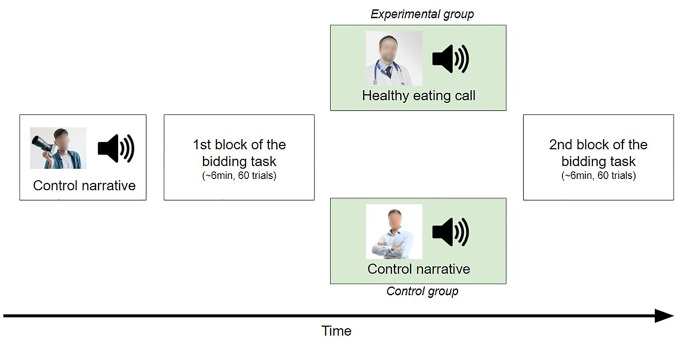
Schematic representation of the experimental procedure.

Three first-person narratives were used in the present study: a “neutral” narrative about photography (duration = 5 min 24 s), a “control” narrative about handwriting (6 min 27 s), and a “healthy eating call” by a nutritionist emphasizing the health risks of sugar consumption (7 min; Figure 2). The latter contained information from scientific sources [e.g., (49)], while the narrator’s personal opinion about sugar consumption was clearly expressed. All of them were written in participants’ native language by us. The audio versions were recorded by different professional male narrators to maximize participants’ engagement. While the narratives were being played, a relevant static image was displayed on the screen (Figure 2). According to the independent cohort’s ratings of emotions after listening to the narratives, the health expert’s narrative (healthy eating call) was found to induce significantly higher fear and sadness as compared to the control narrative, while the effect of the expert’s narratives on the remaining emotions failed to reach significance (Supplementary Table 3).

#### Procedure

At least 2 days prior to attending the experiment, each participant filled out an online demographic questionnaire, stating their gender, age, level of education, occupation and history of any psychological, neurological or metabolic diagnosis. In addition, participants completed the NFC scale [(50, 51) for local language adaptation; the internal consistency in the current study, α = 0.759], as well as the dietary and health knowledge subscale of the Diet and Health Knowledge Survey (DHKS), translated by us (α = 0.787). Participants were asked to not eat anything for at least 3 h prior to the experiment.

Upon arrival at the laboratory, participants saw the real food products to be assured about the validity of the procedure. Then, they performed a practice session, where they had to bid on four products under the same conditions as in the subsequent experimental task. At the beginning of the experiment, all participants listened to a neutral narrative about photography. This aimed to limit the potential caveat that listening to a narrative, regardless of its content, affects food decision-making. Next, they took part in a bidding task, which consisted of 60 trials. Afterward, the experimental group listened to the healthy eating call, whereas the control group listened to a control narrative about handwriting. Finally, all participants performed an additional bidding task, consisting of 60 trials (Figure 2). The stimulus presentation and response recording was controlled by PsychoPy (52).

#### Data analysis

The median scores in the NFC and DHKS scales were used as a cut-off to determine which participants have high or low NFC and health knowledge (26). To test our hypotheses, we estimated four linear-mixed effects models, with subject-level random effects (53). Model 1 aimed to predict the *delta of WTP* (i.e., the WTP for each food product in the second block subtracted by the WTP for the corresponding product in the first block) based on the interaction Group (two levels: experimental, control) × Condition (two levels: sugar-free, sugar- containing), NFC, Health knowledge, Age, Gender and Educational level (four levels: incomplete secondary education, secondary education, incomplete higher education, higher education). Model 2 had the same properties and regressors as Model 1, except that the dependent variable was the delta of reaction time (*delta of RT*, i.e., the RT for each food product in the second block subtracted by the RT for the corresponding product in the first block). Model 3 aimed to predict the delta of WTP only for sugar- containing products based on the interaction Group × NFC, the interaction Group × Health knowledge, Age, Sex and Educational level. Model 4 had the same properties and regressors as Model 3, except that the dependent variable was the delta of WTP only for sugar-free products.

We estimated Models 3 and 4 to explore the moderating role of NFC and health knowledge on Group and delta of WTP, instead of including three-way interactions in Model 1 (i.e., Group × Condition × Trait) for two reasons. First, the statistical power to detect such a three-way interaction would be insufficient (< 80%), as estimated by R’s simr package (54). On the other hand, the statistical power for the predictor Group × Condition was 92% as estimated by the same package. Second, our hypotheses do not apply to such a three-way interaction but rather to the two-way interaction Group × Trait. That is to say, regardless of what the effect of Group is on each condition’s delta of WTP, this effect was expected to be pronounced in participants with high NFC and low health knowledge.

## Results

### Descriptive statistics

The average bid was 44.70 MU (SD = 29.39 MU). Overall, 88.72% of all bids were higher than zero. One-sample Wilcoxon signed rank test showed that the median bid was significantly greater than zero (*W* = 13,058,605, *p* < 0.0001, effect size = 0.857), suggesting that most food products were rewarding for the participants. The mean RT was 1.78 s (SD = 0.93), while participants failed to bid within the 5 s time limit only in 0.89% of the trials. There was no trend in how the WTP changed across time (Supplementary Figure 1). As data from the first block showed, participants bid more for sugar-free than for sugar-containing products (*t* = 2.36, *p* = 0.023, Cohen’s *d* = 0.340) and the RT was higher for sugar-free products (*W* = 350, *p* = 0.014, effect size = 0.341). Furthermore, the distribution of NFC and health knowledge was not significantly different between the two groups (*t* = 0.474, *p* = 0.638, Cohen’s *d* = 0.137 and *t* = 0.963, *p* = 0.341, Cohen’s *d* = 0.278, respectively). Participants’ WTP in the first block was not associated with their NFC or their health knowledge (Supplementary Figure 2).

### The health expert’s narrative decreased individuals’ WTP for sugar-containing food

**FIGURE 3:**
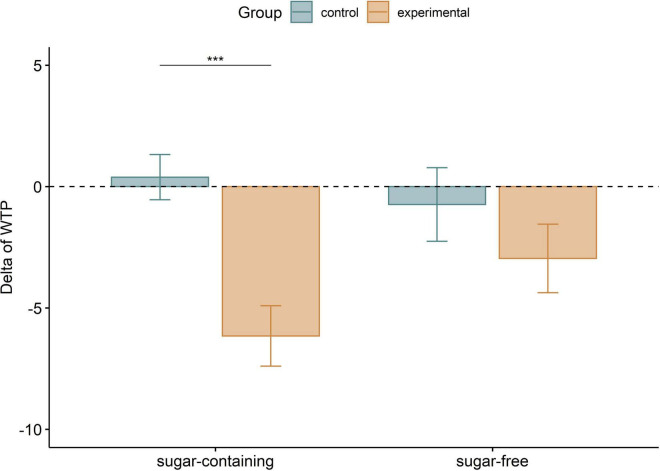
Delta of WTP by group and condition. Due to the problem of *p*-values associated with large samples (70), we first averaged the data of each participant and only then applied pairwise *t*-tests, in order to limit the probability of Type I error. Error bars denote mean ± standard error (SEM), after averaging within each participant. The delta of WTP for sugar-containing products was significantly lower for the experimental group as compared to the control group (*t* = 4.210, Bonferroni corrected *p* = 0.0003, Cohen’s *d* = 1.210), but the delta of WTP for sugar-free products was not statistically different between the two groups (*t* = 1.070, Bonferroni corrected *p* = 0.578, Cohen’s *d* = 0.309). It is worth mentioning that the interaction between Group and Condition on the delta of WTP was also found significant in these averaged data, as assessed by a two-way mixed ANOVA [*F* (1,46) = 5.15, *p* = 0.028], in line with the significant interaction detected by Model 1. ***p < 0.001.

Model 1 aimed to test our hypothesis that the experimental narrative decreases individuals’ WTP for sugar-containing food. Table 1 summarizes the coefficients of this model. The interaction between Condition and Group was statistically significant (*p* < 0.0001). To better understand this interaction effect, we also conducted pairwise t- tests (Figure 3). The results demonstrate that the experimental group decreased their WTP for sugar-containing food products as opposed to the control group. This effect, however, was absent in the sugar-free condition.

**TABLE 1:** Linear mixed effects model for predicting the delta of WTP with subject-level random effects.

Fixed effect	Estimate	SE	95% CI		*P*-value
			LL	UL	
(Intercept)	8.105	5.626	−2.922	19.132	0.158
Gender Male	−0.810	1.973	−4.677	3.057	0.684
Age	−0.194	0.150	−0.489	0.100	0.203
Education Secondary	−7.091	4.827	−16.551	2.369	0.150
Education Incomplete higher	−2.228	5.074	−12.173	7.717	0.663
Education Higher	−2.630	4.895	−12.224	6.964	0.594
NFC High	−1.002	1.952	−4.827	2.823	0.611
DHK High	−0.671	1.880	−4.355	3.013	0.723
Group Experimental	−2.270	1.759	−5.717	1.177	0.203
Condition Sugar-containing	1.125	0.863	−0.567	2.817	0.193
Group Experimental × Condition Sugar-containing	−4.319	1.221	−6.712	−1.926	<0.0001

Since the color of the labels differed across participants, we performed multiple *t*-tests to examine whether the observed effects differed between the label colors, with the results not reaching significance (Supplementary Table 4). Hence, the color of the labels did not confound the data.

Supplementary Table 5 summarizes the coefficients of Model 2, which aimed at predicting the delta of RT. Once again, the interaction between Condition and Group was found to be statistically significant (*p* < 0.0001).

Provided that the interaction effect of Group and Condition was significant both on the delta of WTP and on the delta of RT, we sought to examine whether the latter two measures were correlated with each other. Considering that each participant corresponded to multiple observations in the dataset, we calculated the repeated-measures Pearson’s correlation coefficient (55) between the delta of WTP and delta of RT by group and condition. Correlation values ranged between −0.030 and 0.029, with none of them reaching significance.

### The effect of the health expert’s narrative on individuals’ WTP was not moderated by NFC or health knowledge

Model 3 aimed to assess whether the effect of the expert’s narrative on the WTP for sugar-containing food is moderated by participants’ NFC or health knowledge. Supplementary Table 6 summarizes the coefficients of this model. The interaction between Group and NFC, as well as the interaction between Group and health knowledge on the delta of WTP for sugar-containing products was not statistically significant (*p* = 0.707 and *p* = 0.540, respectively).

Model 4 aimed to assess whether the effect of the expert’s narrative on the WTP for sugar-free food is moderated by participants’ NFC or health knowledge. Supplementary Table 7 summarizes the coefficients of this model. The interaction between Group and NFC, as well as the interaction between Group and health knowledge on the delta of WTP for sugar-free products was not statistically significant (*p* = 0.912 and *p* = 0.753, respectively). Overall, the results demonstrate that the effect of Group on the delta of WTP is not moderated by participants’ NFC or health knowledge (Supplementary Figure 3).

## Discussion

The aim of our work was to further study how healthy eating calls—first-person narratives by health experts—can affect individuals’ willingness to pay for sugar-free and sugar-containing food products. In general, the health expert’s first-person narrative emphasizing the health risks of sugar decreased individuals’ WTP for sugar- containing food, but did not modulate their WTP for sugar-free food. This supports earlier investigations on other healthy eating nudges (e.g., size enhancements), suggesting that interventions are more effective at reducing unhealthy eating than increasing healthy eating (12, 56, 57). This result is also in line with the notion of negativity bias (58, 59). Importantly, the present study extends previous work on nudging healthy food choices through narratives by applying this type of intervention to adults (24, 25). Altogether, the results demonstrate that people tend to conform to first-person narratives when evaluating food products.

While asking the participants of the main study to rate their emotions after listening to the narratives would better address the role of emotions in the observed effects, our pilot’s findings suggest that the pronounced fear and sadness the expert’s narrative induced may have contributed to the reduction of WTP for sugar-containing food. This speculation is supported by a previous field experiment, where graphic warning labels (e.g., tooth decay photos) decreased the share of sugar-containing drinks purchased in a cafeteria (60). In general, negative mood has been associated with greater food intake, although the link between emotions and eating behavior is not yet clear (61–65).

Data from the first block of the bidding task showed the WTP was higher for sugar-free than for sugar-containing products, suggesting that the labels *per se* had an effect on participant’s WTP. This supports the so-far debated hypothesis that sugar- free or reduced-sugar labeling increases individuals’ WTP (16–18). Notably, in our design the label in question was presented with no additional nutrition claims in contrast to previous studies (16, 18), limiting the possibility that the observed effect is confounded by information not related to sugar.

The effect of the expert’s narrative on individuals’ WTP for sugar-containing or sugar-free food was not moderated by their NFC or health knowledge. This contrasts earlier findings showing that both of these traits moderate the effect of narrative messages, but also the effect of statistical messages (in opposite direction), on food product evaluation (26). In our design, the experimental treatment was a complex narrative containing both statistical information and the personal opinion of a health expert. This might have resulted in the phenomena canceling each other out. Moreover, the results of the study in question (26) were based on a single product evaluation, whereas here we utilized 60 different products in order to test our hypotheses without product-specific bias (4, 46, 47, 66).

Certain limitations of this study need to be taken into account. First, the sugar- containing products were labeled as “sugar-containing.” This was done to ensure a clear discrimination between the two conditions, however, there are no labels in the real market highlighting the unhealthy content of food. Second, we investigated only food products which are inexpensive. This limitation is unlikely to be critical, however, since the food category of interest (i.e., sweets) is generally inexpensive. Besides, earlier investigations on food choices have implemented a similar monetary range and even studies where participants could freely bid have reported low average values (4, 46, 47, 66). Third, although we used the educational level, NFC and DHK as measures of our participants’ understanding of the importance of healthy eating, further socio-cultural variables might have influenced the observed effects.

Future research may extend our work by applying first-person narratives by health experts about other categories of unhealthy eating (e.g., high fat) or about the appropriate amount of food intake. Another study could test the importance of the narrator role in our paradigm, be it an expert or an ordinary person. Thus, Dong (67) showed that an expert was most persuasive to people with high health consciousness, while low health conscious people were most influenced by an ordinary person. At the same time, that study revealed that the informative argument was more powerful, when used by an expert compared to an ordinary person. Furthermore, in order to provide a clearer understanding of the mechanisms under which such interventions modulate the WTP for unhealthy food, future studies may employ functional magnetic resonance imaging during listening to the narrative. It would then be promising to conduct an intersubject representational similarity analysis (68) to investigate whether the similarity of brain responses to the narrative predicts the level of conformity to the narrator’s claims in the subsequent decisions.

Taken together, the important contribution of our work to the field of healthy eating nudges is the introduction of first-person narratives by health experts as a type of intervention for improving healthy eating in adults. Moreover, the present study contributes to the debated topic of how emotions affect eating behavior, by suggesting that unhealthy eating might be susceptible to alteration by fear and sadness. Overall, our findings may stimulate novel marketing approaches aiming at assisting consumers in their food choices.

## Data availability statement

The datasets presented in this study can be found in online repositories. The names of the repository/repositories and accession number(s) can be found below: https://osf.io/894mk/ (69).

## Ethics statement

The studies involving human participants were reviewed and approved by National Research University Higher School of Economics. The patients/participants provided their written informed consent to participate in this study.

## Author contributions

IN: conceptualization, methodology, software, validation, formal analysis, investigation, resources, data curation, writing—original draft, visualization, and project administration. KP: conceptualization, methodology, resources, and writing—review and editing. YG: conceptualization, investigation, and resources. AS: conceptualization, methodology, and writing—review and editing. VlK: methodology, resources, and writing—review and editing. IJ: writing—review and editing and funding acquisition. DK and SB: investigation. VaK: conceptualization, methodology, writing—review and editing, supervision, project administration, and funding acquisition. All authors contributed to the article and approved the submitted version.

## Conflict of interest

The authors declare that the research was conducted in the absence of any commercial or financial relationships that could be construed as a potential conflict of interest.

## Publisher’s note

All claims expressed in this article are solely those of the authors and do not necessarily represent those of their affiliated organizations, or those of the publisher, the editors and the reviewers. Any product that may be evaluated in this article, or claim that may be made by its manufacturer, is not guaranteed or endorsed by the publisher.
