# Supplementary material for: “Expert persuasion” can decrease willingness to pay for sugar-containing food
Source: Front Nutr. 2022 Jul 28;9:926875. doi: 10.3389/fnut.2022.926875 (PMC9366858; doi:10.3389/fnut.2022.926875)
Supplement: Supplementary file 1 [file Data_Sheet_1.PDF]

Supplementary Material

Supplementary Figures

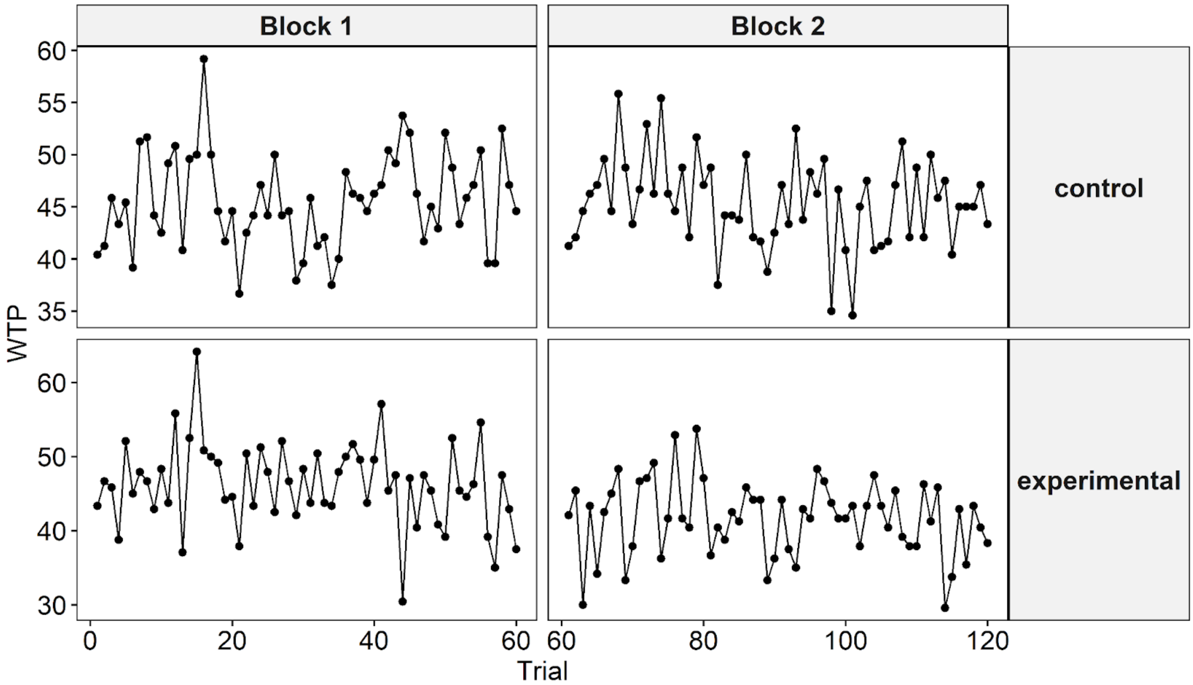

Supplementary Figure 1. Learning effect by group and block. For each group and each block, we calculated the average WTP per trial across participants. This resulted in a time series of 60 time points (since each block consisted of 60 trials). Then, we examined whether the linear trend in these time series is significantly different from zero, by applying a Sieve-bootstrap Student's t-test, as implemented in R's notrend_test() function of the funtimes package. These tests didn't reach significance, suggesting that there was no trend in how participants changed their WTP across time. In fact, the p-values were 0.152, 0.501, 0.336 and 0.162 for the experimental/block 1, control/block 1, experimental/block 2 and control/block 2, respectively.

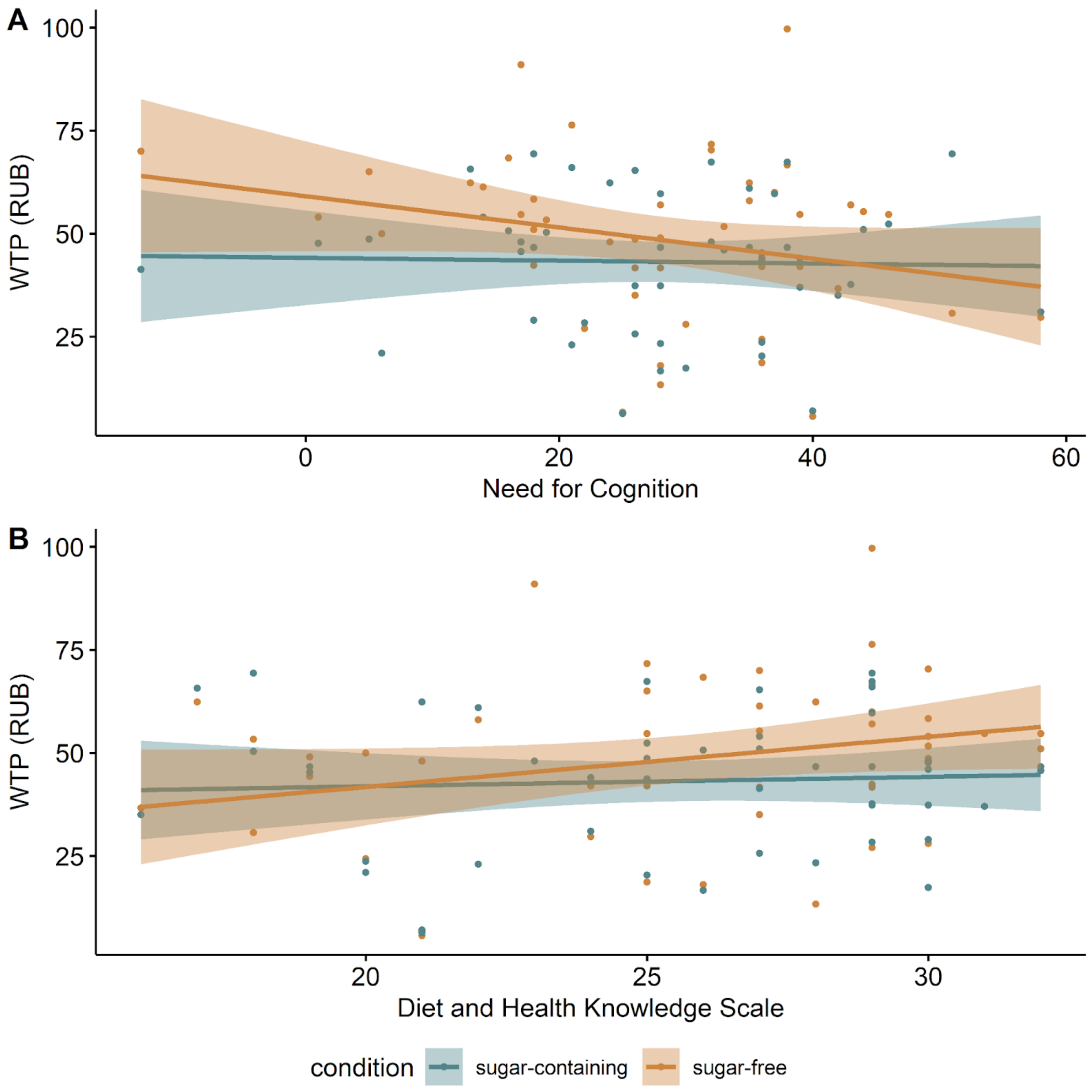

Supplementary Figure 2. Correlation of WTP in the first block with NFC (A) and Health knowledge (B). The Pearson correlation coefficient was not statistically significant ($r = -0.03$, $p = 0.85$ for NFC and WTP for sugar-containing; $r = -0.25$, $p = 0.09$ for NFC and WTP for sugar-free; $r = 0.06$, $p = 0.69$ for health knowledge and WTP for sugar-containing; $r = 0.26$, $p = 0.07$ for health knowledge and WTP for sugar-free).

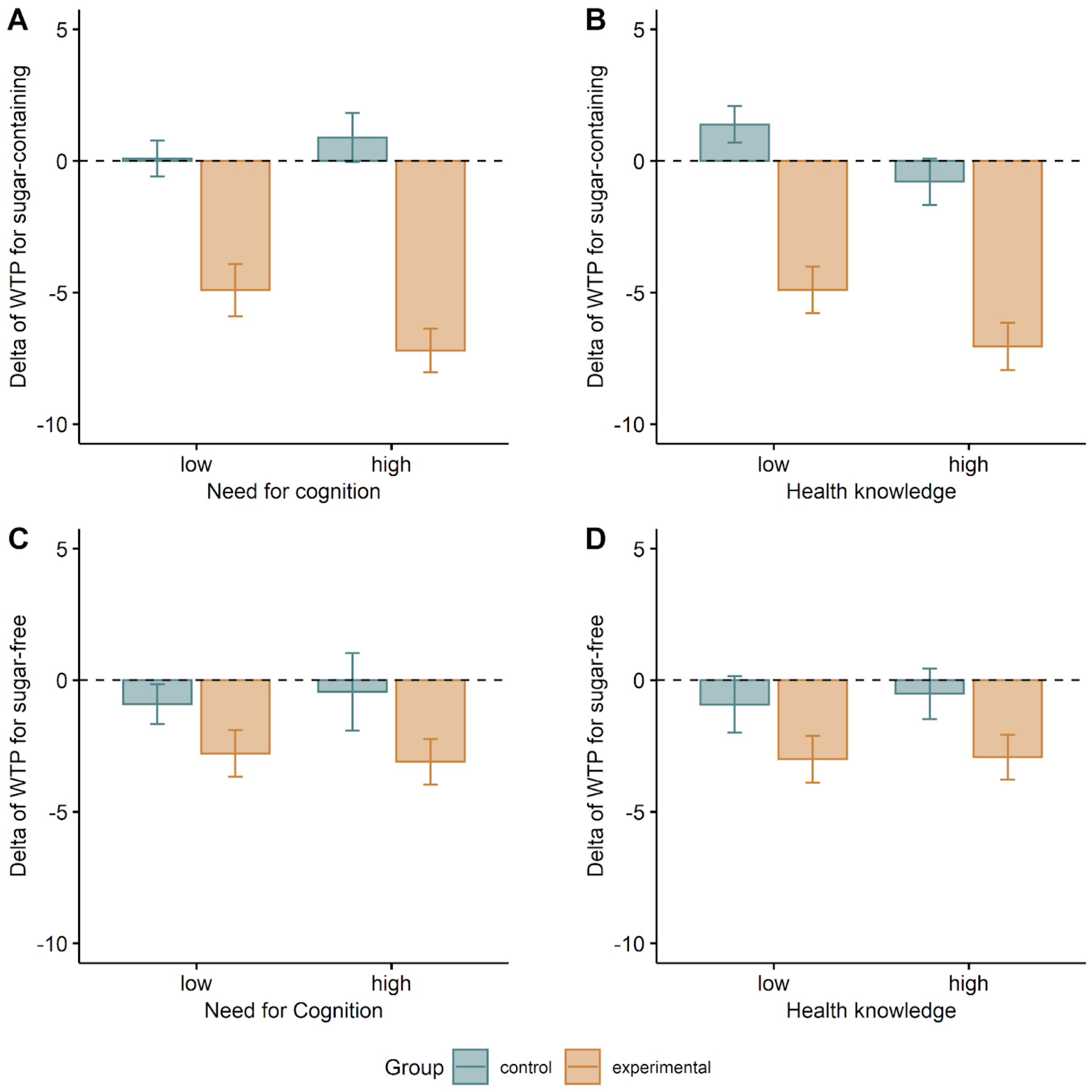

Supplementary Figure 3. *The non-significant moderating effect of NFC and health knowledge on Group and delta of WTP. (A) Interaction between NFC and Group on delta of WTP for sugar-containing products. (B) Interaction between NFC and Group on delta of WTP for sugar-free products. (C) Interaction between health knowledge and Group on delta of WTP for sugar-containing products. (D) Interaction between health knowledge and Group on delta of WTP for sugar-free products. Error bars denote mean ± standard error (SEM).*

Supplementary Tables

Supplementary Table 1

Validation of the food stimuli. Participants were presented with all the 60 food products used in the current experiment, but with no label (i.e. sugar-free or sugar-containing). After the presentation of each product, they had to indicate their WTP on a 0-150 MU scale, their perceived healthfulness (5-point scale), perceived tastefulness (5-point scale), and perceived sweetness (5-point scale). We first extracted the average rating of each participant per condition and then performed a Wilcoxon signed rank test on paired samples.

Variable	Sugar-containing			Sugar-free			W	p-value	Effect size
	N	M	SD	N	M	SD			
WTP	12	51.2	22.3	12	49.7	22.2	60	0.110	0.476
Sweetness	12	3.8	0.4	12	3.8	0.3	42	0.844	0.068
Tastefulness	12	3.4	0.5	12	3.5	0.3	37	0.906	0.045
Healthfulness	12	2.0	0.7	12	2.0	0.8	42	0.450	0.215

Supplementary Table 2

Results of a survey conducted to examine people's opinion about refined sugar and products labelled as "sugar-free". This survey was conducted three months prior to main experiment's data collection. The results are based on the responses of 101 people living in the same city as the participants of the main study. The survey was conducted in participants' native language and here we provide the English translation of it.

- What of the following do you associate with products labelled as "sugar-free"? (multiple choices were possible)<br><br>*a. Health and benefits (49.5%)*<br><br>*b. Low-calorie content (56.4%)*<br><br>*c. Tasteless food (23.8%)*<br><br>*d. Other (2%)*
- What of the following about natural sweeteners (e.g., honey, fructose) do you agree with?<br><br>*a. Natural sweeteners are more useful than refined sugar (51%)*<br><br>*b. Natural sweeteners are not more useful than refined sugar (32%)*<br><br>*c. Natural sweeteners are more harmful than refined sugar (11%)*
- What do you usually try to replace refined sugar with?<br><br>*a. Natural sweeteners (honey, fructose, stevia) (61.9%)*<br><br>*b. Artificial sweeteners (aspartame, sucralose) (23.8%)*<br><br>*c. Other (14.3%)*

Supplementary Table 3

Comparison of emotions the experimental and the control narrative induce. Due to the non-normal distribution of the emotion ratings (Shapiro-Wilk test, p > 0.05), we applied a Mann-Whitney U test for each emotion. P-values were corrected for multiple comparisons with the Benjamini-Hochberg method.

Emotion	Experimental			Control			W	p-value	Effect size
	N	M	SD	N	M	SD			
Valence	20	5.99	0.41	29	6.59	0.37	337	1	0.14
Arousal	20	4.39	0.53	29	4.90	0.37	335	1	0.13
Anger	20	1.69	0.20	29	1.12	0.05	196	0.45	0.27
Joy	20	2.17	0.22	29	2.63	0.25	338	1	0.14
Surprise	20	2.80	0.25	29	2.24	0.25	207	0.75	0.24
Fear	20	2.23	0.30	29	1.07	0.02	102	0.001	0.55
Sadness	20	2.20	0.26	29	1.08	0.03	104	0.001	0.54
Disgust	20	1.51	0.18	29	1.11	0.04	204	0.66	0.25

Supplementary Table 4

Comparison of delta of WTP between the two sets of coloured labels per group and condition. For each participant, we extracted the average delta of WTP for sugar-free products (based on 30 trials) and the average delta of WTP for sugar-containing products (30 trials).

Group	Condition	Set of labels #1			Set of labels #2			T	p-value	Cohen's d
		N	M	SD	N	M	SD			
control	sugar-containing	12	0.47	5.60	12	0.31	3.48	0.09	.93	0.04
control	sugar-free	12	- 0.27	9.70	12	- 0.69	5.19	- 0.03	.98	0.01
experimental	sugar-containing	11	- 6.61	7.00	13	- 5.77	5.50	- 0.32	.75	0.13
experimental	sugar-free	11	- 3.15	4.55	13	- 2.80	8.63	- 0.13	.90	0.05

Supplementary Table 5

Linear mixed effects model for predicting the delta of RT with subject-level random effects.

Fixed Effect	Estimate	SE	95% CI		p-value
			LL	UL	
(Intercept)	0.08	0.293	-0.493	0.654	0.785
Gender Male	-0.212	0.102	-0.413	-0.012	0.045
Age	0.006	0.008	-0.009	0.022	0.410
Education Secondary	-0.221	0.25	-0.712	0.269	0.382
Education Incomplete higher	-0.355	0.263	-0.871	0.161	0.185
Education Higher	-0.397	0.254	-0.895	0.101	0.126
NFC High	0.191	0.101	-0.008	0.389	0.067
DHK High	-0.195	0.098	-0.387	-0.004	0.052
Group Experimental	0.063	0.095	-0.124	0.25	0.511
Condition Sugar-containing	0.124	0.06	0.007	0.241	0.038
Group Experimental x Condition Sugar-containing	-0.213	0.084	-0.378	-0.048	0.012

Supplementary Table 6

Linear mixed effects model for predicting the delta of WTP for sugar-containing products with subject-level random effects.

Fixed Effect	Estimate	SE	95% CI		p-value
			LL	UL	
(Intercept)	2.099	5.811	-9.29	13.488	0.720
Gender Male	-0.523	1.937	-4.319	3.273	0.788
Age	-0.19	0.155	-0.494	0.113	0.226
Education Secondary	-0.277	4.728	-9.544	8.99	0.954
Education Incomplete higher	4.621	4.972	-5.124	14.366	0.359
Education Higher	5.501	4.824	-3.954	14.955	0.261
NFC High	-1.041	2.648	-6.231	4.149	0.697
DHK High	-1.475	2.418	-6.214	3.265	0.546
Group Experimental	-4.924	3.112	-11.024	1.175	0.122
Group Experimental x NFC High	-2.545	3.496	-9.397	4.307	0.471
Group Experimental x DHK High	-0.326	3.297	-6.788	6.136	0.922

Supplementary Table 7

Linear mixed effects model for predicting the delta of WTP for sugar-free products with subject-level random effects.

Fixed Effect	Estimate	SE	95% CI		p-value
			LL	UL	
(Intercept)	13.84	7.864	-1.574	29.254	0.087
Gender Male	-1.227	2.621	-6.365	3.91	0.642
Age	-0.155	0.21	-0.566	0.255	0.463
Education Secondary	-13.833	6.399	-26.374	-1.291	0.037
Education Incomplete higher	-9.007	6.729	-22.196	4.182	0.189
Education Higher	-11.303	6.528	-24.099	1.493	0.092
NFC High	0.848	3.584	-6.176	7.872	0.814
DHK High	0.082	3.273	-6.332	6.497	0.98
Group Experimental	-2.097	4.212	-10.352	6.159	0.622
Group Experimental x NFC High	-0.895	4.731	-10.169	8.378	0.851
Group Experimental x DHK High	-0.078	4.462	-8.823	8.667	0.986
